# Microbiome Research Is Becoming the Key to Better Understanding Health and Nutrition

**DOI:** 10.3389/fgene.2018.00212

**Published:** 2018-06-13

**Authors:** Dirk Hadrich

**Affiliations:** European Commission, Innovative and Personalised Medicine Unit, Health Directorate, Directorate-General Research and Innovation, Brussels, Belgium

**Keywords:** microbiome, health research, personalised medicine, metagenomics, diet, multi-disciplinarity, EU projects, funding opportunities

## Abstract

The human microbiome has emerged as the crucial moderator in the interactions between food and our body. It is increasingly recognised that the microbiome can change our mind and health status, or switch on a wide range of diseases including cancer, cardio-metabolic diseases, allergies, and obesity. The causes of diseases are often only partially understood. However, nutrients, metabolites, and microbes are increasingly regarded as key players, even where the complete disease mechanisms remain unclear. The key to progress in the future will be to use and exploit additional, newly emerging disciplines such as metagenomics to complement patient information and to bring our understanding of diseases and the interrelation and effects of nutritional molecules to the next level. The EU has already funded 216 projects under the 7th Framework Programme and Horizon 2020 programmes to promote metagenomics and to advance our knowledge of microbes. This support started with the catalysing MetaHIT project that has produced a catalogue of gut microbes, and has arrived now at the very multi-disciplinary SYSCID action looking at how the microbiome is driving its resilience potential and our health. Together, these projects involve an investment of more than €498 M. However, in Horizon 2020, the new EU Health and Food Work Programmes for 2018–2020 go even further by setting new goals to find applications and to generate more knowledge on the microbiome, nutrition, various hosts of microbes, and their relation to health and disease. The big vision is to modulate health and diseases via the microbiome and nutrition, while at the same time other factors such as omics, molecular signatures, and lifestyle are constant. In this way, microbiome and nutrition research is moving from an isolated and despised offside position to a beacon of hope with a lot of potential and possibilities.

## Introduction

Personalised medicine aims to develop tailor-made diagnostic, treatment, and prevention approaches using the individual characteristics of patients^1^. It has been pushed by advances in molecular profiling and omics disciplines that allow a deeper understanding and better targeted strategies differing from person to person. Metagenomics has evolved to learn more about the hidden diversity of microbes living with us. Initially, this discipline had the major challenges of sequencing and understanding over 100 trillion bacteria living in the gut and elsewhere in the human body (American Academy of Microbiology, 2013). At the same time, the other omics fields also exploited their own potential and provided mature analytical technologies. However, it soon became obvious that combination and integrative analysis has much more potential and offers many new opportunities. Now, the ongoing digital transformation further drives personalised medicine forward to use artificial intelligence to analyse and find health trends in sets of big data. Citizens also increasingly like to collect their own lifestyle and nutritional data. Integration of these data into the comprehensive health assessment could help to get even more complete health pictures, thus allowing the best treatment and prevention strategies. Metagenomics could become an indispensable interface discipline, telling us about individual interactions between our genes and what we eat. The EU has been committed to metagenomics and microbiome research, with investments of 498 M in 216 projects (Table 1) under the latest two framework programmes for research and technological development, the 7th Framework Programme (FP7)^2^ and Horizon 2020 (H2020)^3^. This article describes the results of these two framework programmes and their projects, many of which are still ongoing. It is interesting to see how this research area has evolved and to analyse its potential. The analysis justifies seamless support in the future under specific funding requirements. As mentioned in the chapter “Working method,” it is important to be aware that even though the funding instrument FP7 finished in 2013, some FP7 projects and many H2020 actions are still ongoing, and some H2020 actions have just started. In many cases, final scientific results and deliverables are pending and expected to be published in scientific journals and on CORDIS^4^. More detailed descriptions of the projects' tasks, objectives, and goals can be found on CORDIS and on the projects' websites.

**Table 1:** Number of EU-funded projects with budget for different research areas and periods.

**Period**		**Health, no nutrition**	**Health and nutrition**	**Health**	**Non-health**	**All microbiome**
2007–2013	No. projects	33	7	40	51	91
(FP7)	€M	118	35.4	153.4	89.6	243
2014–2017	No. projects	58	15	73	52	125
(H2020)	€M	139	28.2	167.2	87.9	255
2007–2017	No. projects	91	22	113	103	216
(FP7,H2020)	€M	257	63.6	320.6	177.5	498

## Basic microbiome research projects: laying the foundation

While the specific roles and microbial interactions of the gut microbiome are mostly unknown, it is well understood that the human gastrointestinal tract houses over 100 trillion bacteria (American Academy of Microbiology, 2013), whose interactions may be essential to human health. MetaHIT^5^ made a breakthrough in this area by assessing and cataloguing comprehensively 3.3 million microbial genes from the human gut-more than 150 times the number of human genes (Qin et al., 2010). As a first step, it was important to know what kind of bacteria are in our gut, to provide a solid foundation for further scientific exploration and knowledge building. MetaHIT researchers proposed that humans have three distinct enterotypes, which are not nation or continent specific (Arumugam et al., 2011). This proposal was controversial; however, MetaHIT researchers thought that the intestinal microbiota variation could be generally stratified with different levels of specific bacteria. Individual host properties such as body mass index, age, or gender could not explain the observed enterotypes. In contrast to the sharply delimited human blood groups, they described the enterotypes as densely populated areas in a multi-dimensional space of community composition. The bioinformatics analysis showed some differences in bacterial species and composition among ulcerative colitis patients, obese patients, and healthy people. Most interesting were the quantitative findings and gene counts for anti- and pro-inflammatory species and links to inflammatory bowel diseases, type 2 diabetes, obesity, and ischaemic cardiovascular disorders (Li et al., 2014). MetaHIT and other large projects across the world generated big metagenomics data sets that were very useful for studying the complexity, variability, and effects of our microbial communities. In this context, it was most important to optimise the comparability of all data at an international level and to launch the coordination support action IHMS (International Human Microbiome Standards)^6^. This developed standard operating procedures and protocols for human sample processing, quality control procedures, using metadata, etc. Collaborations geared toward synthesising current knowledge and building resources to embark on new discoveries at the global level were initiated by the formation of the IHMS^7^. Despite the advances in DNA sequencing, agreement is still lacking on standards and protocols for the comparison of results at global level. This hinders knowledge building. Nevertheless, recently, the Earth Microbiome Project Consortium went further and collected hundreds of microbial samples from researchers worldwide to make a catalogue of the Earth's microbiome and its diversity (Thompson et al., 2017).

Over 6 years, physicians and researchers of METACARDIS^8^ have gathered data from the gut microbiomes of 2000 people. They are still examining 1,400 clinical, biological, and lifestyle variables, along with stool, serum, and urine samples, in an attempt to find biomarkers and risk factors for cardiometabolic diseases including obesity^9^. Metabolic network modeling, metagenomic, transcriptomic, and metabolomic approaches have been used. Based on the impact of changes in gut microbiota and their interaction with the host, it has been proposed that a poor nutritional environment and poor lifestyle choices promote the progression of these diseases. For example, healthier diets can lead to improved cholesterol and inflammatory markers, and weight loss can increase gut-microbial diversity. It also seems that, for some obese people, the surface area of their small intestine can be increased by up to 250%, enabling them to absorb more nutrients; consequently, this can enhance their immune system's inflammatory action in this region (Monteiro-Sepulveda et al., 2015). Although the gut microbiome can be easily influenced by environmental factors, it is an attractive therapeutic target because, theoretically, there is good amenability of the gut microbiome. Therapies could be based on environmental changes, diets, or drugs; however, the challenge will be to ensure effectiveness and to control the dynamic interactions between all factors. Studies have shown that changing a diet dramatically (e.g., the proportion of fibres, proteins, or fats) leads to relatively quick gut microbiome changes, in which around 30–40% of the bacterial strains vary in their abundance (Singh et al., 2017). In this way, a new microbial identity is obtained, until the diet is changed again. Many drugs have also been shown to significantly alter the microbiome (Maier et al., 2018). From this kind of basic research, we should move on toward the translation of clinical discoveries into new diagnostics and preventive approaches. For this translation the lifestyle-microbiota-human health nexus is becoming important to influence societal decision-making (Flandroy et al., 2018).

MyNewGut^10^ is, in addition to cardiometabolic diseases, investigating the endocrine and nervous systems that regulate energy balance, behaviour, anxiety, mood, and other brain functions. This very large consortium aims to clarify how specific species contribute to obesity and whether they could predict obesity. In this context, the interactions of the underlying species with host, diet, environment, and lifestyle determinants are investigated^11^. In order to work toward the prevention of behavioural and mental disorders, the effects of pro- and prebiotics, innovative foods, and semi-personalised products will be tested. Each person has a different proportion of bacterial species and strains in her or his gut, and maybe about a quarter of the microbiota is personal and influenced by our DNA^12^. The latest results suggest that alterations in the early development stage of gut microbiota in infants could influence the immune maturation process and predispose to coeliac disease (Olivares et al., 2018).

The output of this project could in the end also provide consumers with evidence to assess the reliability of claims about foods. Tools for consumers are becoming more important, as the prevalence of nutritional disorders is rising and concerns have been raised about certain food components that could be associated with allergic reactions, dermatitis herpetiformis, autoimmune coeliac disease, or gluten sensitivity. These effects are scientifically not fully understood. However, surprisingly, many more people demand modified food, so that the supply to the market is much higher than the projected number of patients would indicate (Sapone et al., 2012). More people are empirically trying out their own specific diets for a variety of signs and symptoms. However, symptomatic improvements are hard for consumers to evaluate, while clinical variability and the lack of validated biomarkers make evaluation difficult for physicians. Nutrigenomic approaches have brought clarity, e.g., for the coeliac enteropathy characterised by a specific genetic genotype and autoantibodies. Similar nutrition sensitivities could be distinguished from this disease (Leonard et al., 2017).

FunKeyGut^13^ aims to unravel some unknown and diverse network interactions between cooperating and competing microbes and our gut. Such “functional networks” should be partially based on food webs where certain “keystone species” could play major parts and represent ideal targets for manipulating the gut microbiota to improve health or to protect against infection^14^. Researchers on this project aim to elucidate food webs and networks directly *in situ* in the native community. Recently, they speculated that individual bacteria in drinking water cope with specific conditions by simultaneously consuming diverse dissolved molecules of organic matter (Lesaulnier et al., 2017).

INDIGO^15^ analyses and compares the gut microbiomes of patients with Graves' disease (hyperthyroidism) and Graves' orbitopathy (thyroid eye disease), following the hypothesis that the gut microbiome maintains the balance between inflammatory and non-inflammatory bacteria in the gut-associated lymphoid tissue. Using microbes from patients, researchers are trying to modify the gut microbiomes of mice to better understand the pathophysiology, with the ultimate goal of early disease diagnosis and novel interventions^16^. The interaction of gut microbes and nutrients is thought to be crucial in the autoimmune response. To study these interactions, a clinical trial with different dietary interventions is in progress. The project is still ongoing during 2018, but the researchers suggest that understanding the mechanisms leading to loss of tolerance and autoimmune response will also be applicable to other autoimmune disorders^17^.

ALLERGUT^18^ will try to improve the understanding of allergic disorders, from which more than 30% of all Europeans suffer. The aim is to explain the failure to establish mucosal tolerance and identify “core” microbiome signatures or metabolic pathways that favour allergic predisposition^19^. Discovering the relevant molecular mechanisms would offer the opportunity to prevent and treat allergic disorders, even before the onset of symptoms, through personalised approaches. Recently, it was shown that even slight environmental changes modulate the composition of the lung microbiome in early life, whereas adults show higher resilience toward environmental variations (Kostric et al., 2018).

The microbial involvement in the development of Alzheimer's disease is being investigated by AD-gut^20^. The project is based on mouse experiments that indicated a microbial involvement in the development of Alzheimer's specific brain amyloid plaque, leading to the conclusion that the microbiota may contribute to the development of neurodegenerative diseases (Harach et al., 2017). Based on results from faecal transplantation in mice, researchers are now motivated to search for new indirect diagnostic and therapeutic approaches based on gut microbiome modulation through probiotic cocktails. An encapsulation strategy for the efficient delivery of probiotic strains in the gut would represent a very different alternative strategy compared with approaches that directly target the brain^21^.

HUMAN MICROBIOTA^22^ deals with the important impact that the microbiome can have on health through the production of short-chain fatty acids and their interactions with the host immune system. Crucial nutrients for microbes are dietary fibres that are not metabolised by mammalian enzymes in the small intestines. The project aims to develop dietary and nutraceutical strategies to strengthen the dominance of beneficial microbes in the microbiota^23^. These could involve health promoters such as propionate and butyrate or bacteria with an interactive anti-inflammatory effect. However, we need to know more about the glycan metabolism in the human large bowel before these strategies can be fully developed.

AMYLOMICS^24^ is driven by companies aiming to find new robust enzymes for the synthesis or modification of carbohydrates with better digestive or health properties. Their goal is to make an efficient metagenomic platform technology for enzyme screening that can enable “genome walking”^25^. Through this approach, they have identified thousands of novel genes related to starch and carbohydrate processing and selected more than 50 candidate enzymes for further study and biocatalytic optimisation^26^.

The human microbiome also includes fungi, which can play important parts in immune adaptations, fungal diseases, or infections. FUNMETA^27^ is working on an integrated systems biology approach in this area to clarify molecular effects and metabotypes. They are expected to advance metabolomics for diagnosis of fungal diseases and optimise current antifungal therapies and diets^28^. A steady-state dialogue between the host and its microbiota is crucial to maintain local immune homeostasis. Findings so far indicate that targeting microbial dysbiosis through a combination of antibiotics, probiotics, prebiotics, and microbial metabolites is a promising approach for treatment of infections and other human diseases^29^.

Skin microbiomics, genetics, and transcriptomics have been linked by the project MAARS^30^ in order to provide a multi-parameter analysis of allergy and autoimmunity. These diseases could be triggered by microbe-host interactions that regulate adaptive immune responses, inflammatory cascades, and dysbiotic microbial imbalance^31^. Atopic dermatitis and psoriasis create increasing healthcare costs and other burdens for our societies; hence, it is important to understand the role of the skin microbiome in the pathogenesis of these diseases. The Innovative Medicines Initiative (IMI)^32^, which is a public-private partnership between the EU and the European pharmaceutical industry, has decided to build on MAARS and to further support “Genome-Environment Interactions in Inflammatory Skin Disease”. €18.8 M has been budgeted for this topic under a currently open call for proposals and should be provided as a new grant during 2018^33^.

CURE^34^ deals with the respiratory microbiome, which is characteristically imbalanced (dysbiotic) in asthma patients. There may be a reduced abundance of bacteriophages that can naturally control bacterial populations. This project aims to reinstate eubiosis through phage therapy, where the challenge will be to deal with the effects of adding phage mixtures to the complex ecology of the airways^35^. It is expected that wide-scale prevention through immunisation could be achieved for respiratory syncytial virus, with promising prospects for influenza viruses as well, while additional efforts will be needed in the case of rhinoviruses and other respiratory viruses (Papadopoulos et al., 2017).

The training action FABCARB^36^ is looking at dietary triggers such as fermentable carbohydrates or resistant starch that alter the gut microbiome by different fermentation mechanisms. Researchers are keen to find key fermentative species and to uncover the key differences between the nutrigenomic processes in healthy people and in patients suffering from irritable bowel syndrome. There is also considerable interest from wider society, and there is a high potential and need to exploit this basic research for use in education and nutritional advice. The World Health Organization estimates that about 80% of premature heart disease cases, strokes, and cases of type 2 diabetes, and 40% of cancers, could be avoided if the major risk factors for non-communicable diseases, such as unhealthy diets, were eliminated^37^. The European Commission is working to deliver innovative solutions for personalised nutrition advice and/or support that will help consumers to achieve their optimal health and well-being. New projects should deepen our understanding of the drivers of food choice, the food environment, incentives, and other relevant aspects influencing consumers' motivation and behaviour^38^.

RUMINOMICS^39^ was one of the first projects under FP7 that aimed to exploit-omics technologies and to understand how ruminant gut microbiomes are linked with the host's diet and with methane production. A large-scale genetic association study on 1,000 dairy cows was undertaken to find gut microbiome variations, interconnected factors, desirable traits for breeders, and the effects of feed on methane production. Surprisingly, it was found that cows may emit less methane for genetic reasons, but that these animals are also less efficient in producing milk^40^. Nutrition was seen as the main driver of emissions, and certain gut microbes were discovered to be associated with low methane emissions. Following this project, breeders should select high-efficiency ruminants, rather than low-methane ruminants^41^.

The oceans are the largest reservoir of microbiota on the planet. BathyBiome^42^ collected deep-sea mussels from around the world and performed genome and transcriptome sequencing of their microbiomes. They identified genomic and metabolic adaptations of the microbiome to their hosts and to local environmental conditions^43^. The target habitats certainly differed from human living conditions, but the symbiotic interactions that were discovered among microbes, their hosts, and their environment are fascinating, and evidence for direct effects of ocean microbiota on human health has emerged (Wheeler et al., 2012).

## Advanced multi-disciplinary microbiome research

SYSCID^44^ uses a systemic medicine approach to predict chronic inflammatory diseases (inflammatory bowel disease, systemic lupus erythematodes, and rheumatoid arthritis). An advanced multi-omics approach is used to analyse the single-nucleotide polymorphism variome, methylome, transcriptome, gut microbiome, and lifestyle and nutrition to identify and validate shared and unique core disease signatures. Predictive models and disease reprogramming approaches should lead to validated therapeutic tools and new causative therapies^45^. Researchers report a strong relationship between species diversity and ecosystem stability and believe that microbial resilience, the amount of stress that the microbiome can tolerate, crucially comprises resistance, latitude, state of precariousness, and the organisational state or panarchy of the system (Sommer et al., 2017). A full understanding of these components is necessary to be able to achieve therapeutic manipulation of the microbiome. The potential of this multi-disciplinary teamwork approach was highlighted by the discovery that the immune system could react to unhealthy food in a similar way as to a bacterial infection, with short-term inflammation and a long-lasting genetic reprogramming of the immune cells (Christ et al., 2018).

CrUCCial^46^ also deals with Crohn's disease and ulcerative colitis, by functional and molecular characterisation of patients' cellular pathways using integrated multi-omics, immunologic, barrier integrity, and metagenomic methods. Combined with clinical outcomes, they want to develop an index reflecting the proportional contribution of each of the pathogenic mechanisms in a given patient. This index should be tested in a pilot study to see which factor significantly triggers the diseases^47^.

Eat2beNICE^48^ addresses the connectivity among the gut microbiome, the brain, and behavioural disorders, with a specific focus on the links between maladaptive impulsivity and compulsivity and predispositions to antisocial and addictive behaviours. As a characteristic multi-disciplinary project, it goes beyond nutrition compounds and microbes and also assesses epigenetic patterns, socio-economic status, sex, heritability, physical activities, age, and culture. The eventual aim is to characterise the etiologic paths leading to extreme behaviour and promote subsequent policy changes^49^.

MultipleMS^50^ focuses on methods for patient stratification in multiple sclerosis. Using several large clinical cohorts, multiple data types are assessed, including genetic make-up, multi-omics maps, and lifestyle information. The complex interactions between genetic and non-genetic factors produce heterogeneities in patients and diversities of pathophysiology and clinical manifestations, as well as in responses to therapies and disease progression^51^. The full potential of personalised medicine needs in-depth knowledge of distinct pathways and multiple biomarkers from various different angles, delivering a comprehensive picture of the person's current situation. The latest results suggest that multiple sclerosis could be linked to a western lifestyle, nutrition, and the gut microbiome (Berer et al., 2017).

In the aging EU population, there is an increased level of non-alcoholic fatty liver disease, which is also linked to obesity, cardiovascular disease, and type 2 diabetes (Hardy et al., 2016). EPoS^52^ promises to deliver a substantial and definitive atlas of pathophysiological inter-patient liver variations, which will be relevant for the full spectrum of these diseases. A multi-omics approach should be the best way to gain understanding of the biological and environmental factors driving inter-patient variability (Anstee et al., 2016).

The highly interdisciplinary and inter-sectoral training action MiND^53^ aims to educate a new generation of researchers in the field of neurodevelopmental disorders, by combining pharmacological treatments, (epi-)genetics, dietary interventions to influence the microbiome, novel cognitive assessments, and mindfulness training. The ultimate goal is to understand the pathomechanisms of attention-deficit hyperactivity and autism spectrum disorders and, at the same time, to educate a new generation of researchers who are optimally prepared for private sector and academic careers^54^.

Antibiotics have great therapeutic value, but their application has led to gene transfer events in gut bacteria and subsequent antimicrobial resistance. The association between antibiotic consumption and resistance is well documented (Klein et al., 2018). In this context, the increasing consumption in low- and middle-income countries and the rapid increase in the use of last-resort antibiotics are so disturbing that many countries collect and report antibiotic consumption data with the intention to develop control policies. EVOTAR^55^ includes a multi-disciplinary group to discover evolutionary mechanisms and understand spreading of antibiotic resistance in human pathogens. Metagenomic sequencing and functional metagenomic selections are used to map the human reservoir of antibiotic resistance genes (the resistome), to predict gene flow between different reservoirs and predict future resistance trends. The human gut microbiome has also been shown to be sensitive to non-antibiotic drugs. A study screening more than 1,000 marketed, non-antibiotic drugs found that 24% of these drugs could significantly change the composition of the gut microbiome (Maier et al., 2018). Following these findings, more research is needed on drug-microbiome interactions, side-effect control, drug repurposing, and the influence on antibiotic resistance.

The IMI2-project INNODIA^56^ aims to achieve a breakthrough by creating a clinical EU infrastructure to recruit type 1 diabetes patients and collect a diversity of biological samples including blood, plasma, serum, urine, and stools for microbiome analysis. In order to prevent and treat type 1 diabetes, mechanistic studies have been requested to identify bacterial components and metabolites interacting with the immune system (Paun et al., 2017). The large INNODIA network is preparing to obtain access to samples from thousands of patients and high-risk individuals. The samples collected should be available for high-throughput omics screening discovery technologies in the laboratories of academic and industrial partners. The ultimate goal is to establish a “living biobank” of deeply phenotyped individuals, who have also provided consent for recall^57^.

The multi-omics approach of FORECEE^58^ includes genomics, metagenomics, epigenomics, etc., to target four different female cancers. The FORECEE researchers imagine a future where a woman can go for a simple smear test that would tell her about her individual risk of developing any of these cancers in the next 5 or 10 years, so that she could take preventive actions^59^. It is obvious that the development of many cancers cannot exclusively be associated to one or more genetic mutations and that the tumour environment, the body's interaction with environmental factors, lifestyle, and hormonal and reproductive factors must bear further hidden secrets. They are trying to make a risk prediction tool for personalised prevention and for screening recommendations. They encourage women to participate in one of the six EU research clinics so that they get 6,000 volunteers for their project^60^.

The important societal problem of alcohol overuse is addressed by GALAXY^61^. A negative influence of alcohol dependence and associated liver dysfunction on the gut microbiome has been discovered (Leclercq et al., 2014). Researchers expect a significant crosstalk between the gut microbiome and the liver to be involved in the development and progression of alcoholic liver fibrosis. A very multi-disciplinary team of researchers is trying to integrate clinical, multi-omics, and lifestyle information from alcohol over-users and healthy individuals to find disease signatures and new interventional methods to modulate or restore the imbalanced gut microbiome^62^. Probiotic nutritional interventions are interesting but can further expand specific gut bacteria and, for some patients, can lead to other health risks (Dubinkina et al., 2017).

EnteroBariatric^63^ aims to further improve the remarkably effective bariatric surgical treatment that is used to control obesity and type 2 diabetes. Since microbial activities are highly associated with inflammation and cancer, this therapy affects host physiology and colon cancer risk by modulating gut hormone levels and shifting the microbiome. Researchers on this project use a multidisciplinary systems biology approach to develop a phenotypic, molecular, and mechanistic characterisation of the interplay between the host and the gut microbiota^64^. A comparative bariatric surgery approach has shown that changes in the human gut anatomy are reflected in the microbiome and weight-loss-associated microbial metabolites (Ilhan et al., 2017).

FastBio^65^ aims to uncover the molecular signatures of two dietary regimes: first, the diet perturbation associated with the Eastern Orthodox Christian Church, which is characterised by structured abstaining from specific foods for 180–200 days annually; and, second, the unstructured steady-state diet followed by the general population. A multi-omics approach will be used, with integration of all data in order to clarify the effect of both regimes^66^. Recently, an interesting microbiota-fat axis has been described, which is expected to link intermittent fasting-induced microbial shifts with “beiging” of white fat tissue and increased calorie burning (Li et al., 2017).

## Microbiome research focusing on very specific areas

MD-Paedigree^67^ used biomedical omics, imaging, and other data from multiple sources and integrated them all to make an innovative diagnostic decision support tool allowing paediatricians to do similarity search and comparative outcome analysis. Using this tool, they can select a treatment option and receive on-the-spot support in predicting the likely outcome based on each patient's personal medical data. This allows them to identify hidden patterns; in addition, patients can get in touch with others with similar characteristics. Metagenomic studies involving arthritis patients and obese children have been used to analyse disease susceptibilities and immune responses^68^. Based on the host-gut microbiota interplay, it has been proposed that ecological dynamics and functional alterations of gut microbiota are triggering factors for inflammatory bowel disease and irritable bowel syndrome. Tracking of these triggers would enable the identification of disease profiles, biomarkers, and risk predictors for these diseases (Putignani et al., 2016).

The training action ELITE^69^ looks at evolutionary adaptations that drive biodiversity and natural selection processes. Biological material from ancient Siberian Yakuts is being investigated with multi-omics technologies to learn about possible transitions and maladaptations to our modern lifestyle that make us more susceptible to diseases. Common immune-mediated diseases may be partly caused by evolutionary microbial adaptations, for example, decreased gut microbiome diversity in residents of developed countries may alter mucosal immune responses (Karlsson et al., 2014).

MIND THE GUT^70^ is another training action to analyse the mummified ancient microbiomes of the aboriginal Canary islanders and to clarify the role of the microbiome in overcoming the challenges of diet, environment, and lifestyle changes^71^. Not many efforts have been made so far to investigate how the human gut-systems have changed over history, and whether the microbiome evolution reflects major events of human history.

The large multi-disciplinary EU partnership of 48 organisations LIVESEED^72^ is investigating plant health and exploring plant-microbe interactions with the aim of strengthening sustainable food production and improving breeding efficiency. This project does not directly contribute to health research, but better understanding of the optimal conditions for good food, innovative solutions, and sustainable agriculture are important for our future well-being^73^.

## Conclusions and way forward for EU funding

The EU has funded 216 projects as part of FP7 and H2020 to promote metagenomics and to advance our knowledge of microbes (Table 1). The area has become more important over the years, with more and more projects being funded. Even though H2020 has only been in progress for 3 years, it already includes more projects than FP7. Of these, 113 projects are in the area of health research, and 22 also deal with nutritional aspects; 103 projects are not directly related to health and target areas such as the agri-food sector, nutrition, animals, plants, the marine environment, or the evolution of bacteria. Together, these projects involve an investment of more than €498 M. At a time when the microbiome was unexplored, the first research step was of course to develop and exploit the new metagenomics technologies with the compilation of gene catalogues of microbes. Mature technologies were built up, providing us with more detail about the microbes with which we are living in symbiosis. Without such catalogues and overview, we would not sufficiently know what is present, and research could not deliver meaningful outcomes. Later came an integration of metagenomics within multi-disciplinary approaches that can provide us with more information about the patient. There is a clear trend to go beyond metagenomics and to deepen our understanding through a combination of metagenomics with various other disciplines. This trend also developed as the microbiome emerged as very dynamic, changing, and adaptable. Many studies have shown that the composition as well as the functions of the microbiome can change depending on numerous variables, such as host behaviour, hygiene, use of drugs, habits, lifestyle, genetics, and nutrition. Simultaneously, research has delivered more and more evidence for associations between the microbiome and a wide range of different diseases. Therefore, future research needs to fully clarify the role of all variables, the dependencies, and the interplay between microbiome, nutrition, and host.

Concerning nutrition and lifestyle, we live in a time when society and politicians are keen to further investigate, clarify, and exploit the roles of these variables with regard to health. This demand or desire is continuously nourished by interesting scientific facts that attract public attention, e.g., the publication of results from the French NutriNet-Santé cohort showing associations between ultra-processed food intake and overall risk of breast, prostate, and colorectal cancers (Fiolet et al., 2018).

H2020 aims to address societal challenges, and to deliver smart and sustainable solutions that will overcome those challenges. In the area of health, it aims to translate new knowledge into innovative applications, making health services more efficient and integrating effective personalised medicine approaches. The Health Work Programme of the European Commission for 2018–2020 includes topic BHC-03^74^ to exploit research outcomes and application potential of the human microbiome for personalised prediction and prevention of diseases. This should help to provide more knowledge on the microbiome, its functionalities, healthy conditions, diets, and resilience. Ultimately, novel clinical tools and useful applications should be generated, because the population requires more personalised and effective care. Multi-disciplinarity and digital combinations are the principles to achieve this more diversified care. A budget of €50 M is available to fund several projects under this topic.

The Food, Agriculture and Bioeconomy Work Programme of the European Commission for 2018–2020 contains a cluster of complementary topics on microbiome applications for sustainable food systems, personalised nutrition, coordinating microbiome research, soil biodiversity and interaction with plants, microbiome in aquaculture, and microbiomes influencing terrestrial livestock health^75^.

The Innovative Medicines Initiative (IMI), which is a public-private partnership between the EU and the European pharmaceutical industry, currently has two topics open for which proposals are expected to be submitted and implemented during 2018: “Genome-Environment Interactions in Inflammatory Skin Disease” and “Human tumour microenvironment immunoprofiling.” Both topics cover microbiome research within a very multi-disciplinary spectrum to deepen and widen understanding of the disease. €18.8 M and €27.8 M are budgeted for these topics and should be available in the form of new grants during 2018^76^.

## Future perspectives

Research on the microbiome has delivered many interesting basic findings and advanced technologies, enabling this discipline to move from a marginalised position to become a beacon of hope with great potential and many possibilities.

The field has spread out in many directions, with microbes being studied from various perspectives and sub-fields of different specificities being developed. The International Human Microbiome Consortium will certainly remain an important forum to present data, findings, and views. Increased cooperation and coordination at a global level generally leads to more efficient use of resources and avoids multiple funding. It would be very effective to obtain a consensus on the most important societal priority targets for which scientific development could be fruitful, so that the use of financial and human resources could be concentrated. Faster progress and clear added value can be achieved through better comparability of data, which will require more harmonised and widely agreed standards and protocols. In this respect, a laboratory manual describing in detail the metagenomics technologies and methods would be helpful as a definitive technical source; this would also make the field easily accessible to the wide range of life science disciplines.

The fact that microbes are capable of colonising almost every niche of the human body in symbiosis suggests that they must possess particular outbalanced adaptation mechanisms, the breakdown of which may result in fatal consequences or disease. This breakdown could be detected with diagnostic tools for early prediction and prevention of diseases, e.g., the early diagnosis of type 2 diabetes was shown to be possible prior to clinical onset of the disease (Yassour et al., 2016). Such diagnostic tools could also be very valuable for obesity, which is associated with various diseases and seen as one of the most serious public health problems of our century. Changes in the composition of the microbiome and its diversity have been linked to obesity (Turnbaugh et al., 2006), and predictive diagnostic tools can be expected in the near future.

The ultimate vision in health research is to be able to modulate states of health and disease. This may not be so difficult to achieve in cases where the balanced communication of the microbiome between food and the human body is disrupted, leading to imbalances and diseases such as ulcerative colitis or Crohn's disease. New microbial therapeutic approaches could involve drug- or food-incorporating microbes that could cure diseases.

## Working method

For this article, projects under the funding instruments FP7 and H2020 have been analysed. This covers a time period from 2007 until 2020. However, additional relevant grants may be prepared and implemented during 2018–2020.

Only some of the 216 identified projects have been selected and described in detail to explain diversity, developments, trends, and approaches.

All projects can be found using the CORDIS search engine, at least with abstracts and partners, and often also with annual reports or news^4^. Acronyms together with project IDs should be used to find the projects in CORDIS.

In addition, project websites, internal European Commission data, and project monitoring work have been exploited for this analysis.

Crucial keywords (e.g., microbiome, metagenomics, or microbe) have been clearly identified in the goals, abstract, or objectives of the projects.

Only projects with significant work on metagenomics, microbiome, and microbes have been taken into account. However, it is possible that there are further projects that make contributions to the area analysed.

Projects may have been modified or could be modified in future following the legal amendment procedure.

FP7 ended in 2013, but some FP7 projects and many H2020 actions are still ongoing.

The eventual success of projects and their results also depend on the validation work performed toward the end of the project duration or even afterwards. Final scientific results and deliverables are often pending and expected to be published in the future in scientific journals and on CORDIS.

Lists of FP7 and/or H2020 project acronyms can be provided upon request for specific research areas.

## Author contributions

The author confirms being the sole contributor of this work and approved it for publication.

### Conflict of interest statement

The author declares that the research was conducted in the absence of any commercial or financial relationships that could be construed as a potential conflict of interest.

## Footnotes

^1^European Council. *Council Conclusions on Personalised Medicine for Patients*. Available online at: http://data.consilium.europa.eu/doc/document/ST-15054-2015-INIT/en/pdf (December 7, 2015).

^2^European Commission website, 7^th^ Framework Programme (FP7). Available online at: https://ec.europa.eu/research/fp7/index_en.cfm.

^3^European Commission website, Horizon 2020 (H2020). Available online at: https://ec.europa.eu/programmes/horizon2020/.

^4^European Commission, Community Research and Development Information Service CORDIS, *Public Repository and Portal to Disseminate Information*. Available online at: https://cordis.europa.eu/home_en.html.

^5^FP7 project MetaHIT, Metagenomics of the Human Intestinal Tract, ID: 201052.

^6^FP7 project IHMS, International Human Microbiome Standards, ID: 261376.

^7^Website project IHMS. Available online at: http://www.human-microbiome.org/.

^8^FP7 project METACARDIS, Metagenomics in Cardiometabolic Diseases, ID: 305312.

^9^Website project METACARDIS. Available online at: http://www.metacardis.net/.

^10^FP7 project MyNewGut, Microbiome Influence on Energy balance and Brain Development-Function Put into Action to Tackle Diet-related Diseases and Behavior, ID: 613979.

^11^Website project MyNewGut. Available online at: http://www.mynewgut.eu/.

^12^European Commission, CORDIS Information. Available online at: https://cordis.europa.eu/news/rcn/136017_en.html.

^13^H2020 project FunKeyGut, Illuminating Functional Networks and Keystone Species in the Gut, ID: 714623.

^14^European Commission, CORDIS information: Available online at: https://cordis.europa.eu/project/rcn/209703_de.html.

^15^FP7 project INDIGO, Investigation of Novel biomarkers and Definition of the role of the microbiome In Graves' Orbitopathy, ID: 612116.

^16^European Commission, CORDIS information. Available online at:https://cordis.europa.eu/project/rcn/188639_en.html.

^17^European Commission, CORDIS information. Available online at:https://cordis.europa.eu/result/rcn/193091_en.html.

^18^H2020 project ALLERGUT, Mucosal Tolerance and Allergic Predisposition: Does it all start in the gut?, ID: 716718.

^19^Helmholtz Zentrum München, German Research Center for Environmental Health. Available online at: https://www.helmholtz-muenchen.de/comi/research-unit/news/news/article/40503/index.html.

^20^H2020 project AD-gut, Alzheimer Disease - gut connection, ID: 686271.

^21^Website project AD-gut. Available online at: http://adgut.eu/.

^22^FP7 project HUMAN MICROBIOTA, Understanding and exploiting complex glycan metabolism in the human microbiota, ID: 322820.

^23^European Commission, CORDIS information. Available online at: https://cordis.europa.eu/result/rcn/178297_en.html.

^24^FP7 project AMYLOMICS, Amylol Enzymes Captured by Targeted Metagenomics, ID: 265992.

^25^European Commission, CORDIS information. Available online at: https://cordis.europa.eu/result/rcn/157198_en.html.

^26^European Commission, CORDIS information. Available online at: https://cordis.europa.eu/result/rcn/91746_en.html.

^27^FP7 project FUNMETA, Metabolomics of fungal diseases: a systems biology approach for biomarkers discovery and therapy, ID: 293714.

^28^European Commission, CORDIS information. Available online at: https://cordis.europa.eu/result/rcn/179578_en.html.

^29^European Commission, CORDIS information. Available online at: https://cordis.europa.eu/result/rcn/203851_en.html.

^30^FP7 project MAARS, Microbes in Allergy and Autoimmunity Related to the Skin, ID: 261366.

^31^European Commission, CORDIS information. Available online at: https://cordis.europa.eu/result/rcn/91613_en.html.

^32^Innovative Medicines Initiative (IMI), EU public-private partnership funding health research and innovation. Available online at: https://www.imi.europa.eu/.

^33^Innovative Medicines Initiative (IMI), Topic 2 of IMI2 - Call 13, Call ID: H2020-JTI-IMI2-2017-13-two-stage. Available online at: https://www.imi.europa.eu/apply-funding/open-calls/imi2-call-13.

^34^H2020 project CURE, Constructing a “Eubiosis Reinstatement Therapy” for Asthma, ID: 767015.

^35^European Commission, CORDIS information. Available online at: https://cordis.europa.eu/project/rcn/211920_en.html.

^36^H2020 project FABCARB, Fermentation and behaviour of carbohydrates in the colon, ID: 747438.

^37^World Health Organisation Fact File. Available online at: http://www.who.int/features/factfiles/noncommunicable_diseases/facts/en/index9.html.

^38^European Commission Horizon 2020 Work Programme 2018-2020 *9. Food Security, Sustainable Agriculture and Forestry, Marine, Maritime and Inland Water Research and the Bioeconomy*, Call DT-SFS-14-2018, page 28. Available online at: http://ec.europa.eu/research/participants/data/ref/h2020/wp/2018-2020/main/h2020-wp1820-food_en.pdf.

^39^FP7 project RUMINOMICS, Connecting the animal genome, gastrointestinal microbiomes and nutrition to improve digestion efficiency and the environmental impacts of ruminant livestock production, ID: 289319.

^40^European Commission, CORDIS information. Available online at: https://cordis.europa.eu/result/rcn/92194_en.html.

^41^European Commission, CORDIS information. Available online at: https://cordis.europa.eu/result/rcn/191776_en.html.

^42^FP7 project BathyBiome, The Symbiome of Bathymodiolus Mussels from Hydrothermal Vents: From the Genome to the Environment, ID: 340535.

^43^European Commission, CORDIS information. Available online at: https://cordis.europa.eu/result/rcn/191618_en.html.

^44^H2020 project SYSCID, A Systems medicine approach to chronic inflammatory disease, ID: 733100.

^45^European Commission, CORDIS information. Available online at: https://cordis.europa.eu/news/rcn/137735_en.html.

^46^H2020 project CrUCCial, Novel diagnostic and therapeutic approach to inflammatory bowel disease based on functional characterization of patients: the CrUCCial index, ID: 694679.

^47^European Commission, CORDIS information. Available online at: https://cordis.europa.eu/project/rcn/204842_en.html.

^48^H2020 project Eat2beNICE, Effects of Nutrition and Lifestyle on Impulsive, Compulsive, and Externalizing behaviours, ID: 728018.

^49^Radboud University Medical Center. Available online at: https://www.radboudumc.nl/en/news/2017/een-kookboek-voor-gezond-gedrag.

^50^H2020 project MultipleMS, Multiple manifestations of genetic and non-genetic factors in Multiple Sclerosis disentangled with a multi-omics approach to accelerate personalised medicine, ID: 733161.

^51^Website project MultipleMS. Available online at: https://www.multiplems.eu/.

^52^H2020 project EPoS, Elucidating Pathways of Steatohepatitis, ID: 634413.

^53^H2020 project MiND, Mastering skills in the training Network for attention deficit hyperactivity and autism spectrum Disorders, ID: 643051.

^54^Website project MiND. Available online at: http://mind-project.eu/.

^55^FP7 project EVOTAR, Evolution and Transfer of Antibiotic Resistance, ID: 282004.

^56^H2020 project INNODIA, Translational approaches to disease modifying therapy of type 1 diabetes: an innovative approach towards understanding and arresting type 1 diabetes, ID: 115797.

^57^Innovative Medicines Initiative website information. Available online at: https://www.imi.europa.eu/projects-results/project-factsheets/innodia.

^58^H2020 project FORECEE, Female cancer prediction using cervical omics to individualise screening and prevention, ID: 634570.

^59^European Commission, CORDIS information. Available online at: https://cordis.europa.eu/result/rcn/201993_en.html.

^60^Website project FORECEE. Available online at: https://forecee.eu/.

^61^H2020 project GALAXY, Gut-and-liver axis in alcoholic liver fibrosis, ID: 668031.

^62^Website project GALAXY. Available online at: http://www.livergalaxy.eu/.

^63^H2020 project EnteroBariatric, Investigating Host-Microbial Interactions after Bariatric Surgery, ID: 715662.

^64^European Commission, CORDIS information. Available online at: https://cordis.europa.eu/project/rcn/206321_en.html.

^65^H2020 project FastBio, A genomics and systems biology approach to explore the molecular signature and functional consequences of long-term, structured fasting in humans, ID: 716998.

^66^European Commission, CORDIS information. Available online at: https://cordis.europa.eu/project/rcn/210912_en.html.

^67^FP7 project MD-Paedigree, Model-Driven European Paediatric Digital Repository, ID: 600932.

^68^Website project MD-Paedigree. Available online at: http://www.md-paedigree.eu/.

^69^H2020 project ELITE, Exploring LIfestyle Transitions: a molecular and physical anthropology approach of ancient Yakut people, ID: 748122.

^70^H2020 project MIND THE GUT, Molecular Markers of Microbiome Evolution, ID: 750860.

^71^European Commission, CORDIS information. Available online at: https://cordis.europa.eu/project/rcn/209021_en.html.

^72^H2020 project LIVESEED, Improve performance of organic agriculture by boosting organic seed and plant breeding efforts across Europe, ID: 727230.

^73^Website project LIVESEED. Available online at: https://www.liveseed.eu/.

^74^European Commission Horizon 2020 Work Programme 2018-2020 *8. Health, Demographic Change and Wellbeing*, Call SC1-BHC-03-2018, page 12. Available online at: http://ec.europa.eu/research/participants/data/ref/h2020/wp/2018-2020/main/h2020-wp1820-food_en.pdf.

^75^European Commission Horizon 2020 Work Programme 2018-2020 *9. Food Security, Sustainable Agriculture and Forestry, Marine, Maritime and Inland Water Research and the Bioeconomy*, Calls LC-SFS-03-2018, DT-SFS-14-2018, SFS-32-2018, SFS-01-2018-2019-2020, DT-BG-04-2018-2019, SFS-02-2020. Available online at: http://ec.europa.eu/research/participants/data/ref/h2020/wp/2018-2020/main/h2020-wp1820-food_en.pdf.

^76^Innovative Medicines Initiative (IMI), Topics 2 and 8 of IMI2 - Call 13, Call ID: H2020-JTI-IMI2-2017-13-two-stage. Available online at: https://www.imi.europa.eu/apply-funding/open-calls/imi2-call-13.

## References

[B1] American Academy of Microbiology (2013). Human Microbiome FAQ. Available online at: https://www.asm.org/images/stories/documents/FAQ_Human_Microbiome.pdf

[B2] Anstee Q. M. Seth D. Day C. P. (2016). Genetic factors that affect risk of alcoholic and nonalcoholic fatty liver disease. Gastroenterology 150, 1728–1744. 10.1053/j.gastro.2016.01.037 26873399

[B3] Arumugam M. Raes J. Pelletier E. Le Paslier D. Yamada T. Mende D. R.. (2011). Enterotypes of the human gut microbiome. Nature 473, 174–180. 10.1038/nature09944 21508958 PMC3728647

[B4] Berer K. Gerdes L. A. Cekanaviciute E. Jia X. Xiao L. Xia Z.. (2017). Gut microbiota from multiple sclerosis patients enables spontaneous autoimmune encephalomyelitis in mice. Proc. Natl. Acad. Sci. U.S.A. 114, 10719–10724. 10.1073/pnas.1711233114 28893994 PMC5635914

[B5] Christ A. Günther P. Lauterbach M. A. R. Duewell P. Biswas D. Pelka K.. (2018). Western diet triggers NLRP3-dependent innate immune reprogramming. Cell 172, 162–175. 10.1016/j.cell.2017.12.013 29328911 PMC6324559

[B6] Dubinkina V. B. Tyakht A. V. Odintsova V. Y. Yarygin K. S. Kovarsky B. A. Pavlenko A. V.. (2017). Links of gut microbiota composition with alcohol dependence syndrome and alcoholic liver disease. Microbiome 5:141. 10.1186/s40168-017-0359-2 29041989 PMC5645934

[B7] Fiolet T. Srour B. Sellem L. Kesse-Guyot E. Allès B. Méjean C.. (2018). Consumption of ultra-processed foods and cancer risk: results from NutriNet-Santé prospective cohort. BMJ 360:k322. 10.1136/bmj.k322 29444771 PMC5811844

[B8] Flandroy L. Poutahidis T. Berg G. Clarke G. Dao M. C. Decaestecker E.. (2018). The impact of human activities and lifestyles on the interlinked microbiota and health of humans and of ecosystems. Sci. Total Environ. 627, 1018–1038. 10.1016/j.scitotenv.2018.01.288 29426121

[B9] Harach T. Marungruang N. Duthilleul N. Cheatham V. Mc Coy K. D. Frisoni G.. (2017). Reduction of Abeta amyloid pathology in APPPS1 transgenic mice in the absence of gut microbiota. Sci. Rep. 7:41802. 10.1038/srep41802 28176819 PMC5297247

[B10] Hardy T. Oakley F. Anstee Q. M. Day C. P. (2016). Nonalcoholic fatty liver disease: pathogenesis and disease spectrum. Annu. Rev. Pathol. 11, 451–496. 10.1146/annurev-pathol-012615-044224 26980160

[B11] Ilhan Z. E. DiBaise J. K. Isern N. G. Hoyt D. W. Marcus A. K. Kang D.-W.. (2017). Distinctive microbiomes and metabolites linked with weight loss after gastric bypass, but not gastric banding. ISME J. 11, 2047–2058. 10.1038/ismej.2017.71 28548658 PMC5563958

[B12] Karlsson E. K. Kwiatkowski D. P. Sabeti P. C. (2014). Natural selection and infectious disease in human populations. Nat. Rev. Genet. 15, 379–393. 10.1038/nrg3734 24776769 PMC4912034

[B13] Klein E. Y. Van Boeckel T. P. Martinez E. M. Pant S. Gandra S. Levin S. A.. (2018). Global increase and geographic convergence in antibiotic consumption between 2000 and 2015. Proc. Natl. Acad. Sci. U.S.A. 115, E3463–E3470. 10.1073/pnas.1717295115 29581252 PMC5899442

[B14] Kostric M. Milger K. Krauss-Etschmann S. Engel M. Vestergaard G. Schloter M.. (2018). Development of a stable lung microbiome in healthy neonatal mice. Microb. Ecol. 75, 529–542. 10.1007/s00248-017-1068-x 28905200

[B15] Leclercq S. Matamoros S. Cani P. D. Neyrinck A. M. Jamar F. Stärkel P.. (2014). Intestinal permeability, gut-bacterial dysbiosis, and behavioral markers of alcohol-dependence severity. Proc. Natl. Acad. Sci. U.S.A. 111, 4485–4493. 10.1073/pnas.1415174111 25288760 PMC4210345

[B16] Leonard M. M. Sapone A. Catassi C. Fasano A. (2017). Celiac disease and nonceliac gluten sensitivity: a review. JAMA 318, 647–656. 10.1001/jama.2017.9730 28810029

[B17] Lesaulnier C. C. Herbold C. W. Pelikan C. Berry D. Gérard C. Le Cozet X.. (2017). Bottled aqua incognita: microbiota assembly and dissolved organic matter diversity in natural mineral waters. Microbiome 5:126. 10.1186/s40168-017-0344-9 28938908 PMC5610417

[B18] Li G. Xie C. Lu S. Nichols R. G. Tian Y. Li L.. (2017). Intermittent fasting promotes white adipose browning and decreases obesity by shaping the gut microbiota. Cell Metab. 26, 672–685. 10.1016/j.cmet.2017.08.019 28918936 PMC5668683

[B19] Li J. Jia H. Cai X. Zhong H. Feng Q. Sunagawa S.. (2014). An integrated catalog of reference genes in the human gut microbiome. Nat. Biotechnol. 32, 834–841. 10.1038/nbt.2942 24997786

[B20] Maier L. Pruteanu M. Kuhn M. Zeller G. Telzerow A. Anderson E. E.. (2018). Extensive impact of non-antibiotic drugs on human gut bacteria. Nature 555, 623–628. 10.1038/nature25979 29555994 PMC6108420

[B21] Monteiro-Sepulveda M. Touch S. Mendes-Sá C. André S. Poitou C. Allatif O.. (2015). Jejunal T cell inflammation in human obesity correlates with decreased enterocyte insulin signaling. Cell Metab. 22, 113–124. 10.1016/j.cmet.2015.05.020 26094890

[B22] Olivares M. Walker A. W. Capilla A. Benítez-Páez A. Palau F. Parkhillet J.. (2018). Gut microbiota trajectory in early life may predict development of celiac disease. Microbiome 6:36. 10.1186/s40168-018-0415-6 29458413 PMC5819212

[B23] Papadopoulos N. G. Megremis S. Kitsioulis N. A. Vangelatou O. West P. Xepapadaki P. (2017). Promising approaches for the treatment and prevention of viral respiratory illnesses. J. Allergy Clin. Immunol. 140, 921–932. 10.1016/j.jaci.2017.07.001 28739285 PMC7112313

[B24] Paun A. Yau C. Danska J. S. (2017). The Influence of the Microbiome on Type 1 Diabetes. J. Immunol. 198, 590–595. 10.4049/jimmunol.1601519 28069754

[B25] Putignani L. Del Chierico F. Vernocchi P. Cicala M. Cucchiara S. Dallapiccola B.. (2016). Gut microbiota dysbiosis as risk and premorbid factors of IBD and IBS along the childhood-adulthood transition. Inflamm. Bowel Dis. 22, 487–504. 10.1097/MIB.0000000000000602 26588090

[B26] Qin J. Li R. Raes J. Arumugam M. Burgdorf K. S. Manichanh C.. (2010). A human gut microbial gene catalogue established by metagenomic sequencing. Nature 464, 59–65. 10.1038/nature08821 20203603 PMC3779803

[B27] Sapone A. Bai J. C. Ciacci C. Dolinsek J. Green P. H. R. Hadjivassiliou M.. (2012). Spectrum of gluten-related disorders: consensus on new nomenclature and classification. BMC Med. 10:13. 10.1186/1741-7015-10-13 22313950 PMC3292448

[B28] Singh R. K. Chang H. W. Yan D. Lee K. M. Ucmak D. Wong K.. (2017). Influence of diet on the gut microbiome and implications for human health. J. Transl. Med. 15:73. 10.1186/s12967-017-1175-y 28388917 PMC5385025

[B29] Sommer F. Anderson J. M. Bharti R. Raes J. Rosenstiel P. (2017). The resilience of the intestinal microbiota influences health and disease. Nature 15, 630–638. 10.1038/nrmicro.2017.58 28626231

[B30] Thompson L. R. Sanders J. G. McDonald D. Amir A. Ladau J. Locey K. J.. (2017). A communal catalogue reveals Earth's multiscale microbial diversity. Nature 551, 457–463. 10.1038/nature24621 29088705 PMC6192678

[B31] Turnbaugh P. J. Ley R. E. Mahowald M. A. Magrini V. Mardis E. R. Gordon J. (2006). An obesity-associated gut microbiome with increased capacity for energy harvest. Nature 444, 1027–1031. 10.1038/nature05414 17183312

[B32] Wheeler B. W. White M. Stahl-Timmins W. Depledge M. H. (2012). Does living by the coast improve health and wellbeing? Health Place 18, 1198–1201. 10.1016/j.healthplace.2012.06.015 22796370

[B33] Yassour M. Lim M. Y. Yun H. S. Tickle T. L. Sung J. Song Y.-M.. (2016). Sub-clinical detection of gut microbial biomarkers of obesity and type 2 diabetes. Genome Med. 8:17. 10.1186/s13073-016-0271-6 26884067 PMC4756455

